# Co-occurrence of *Artemisia* and *Ambrosia* pollen seasons against the background of the synoptic situations in Poland

**DOI:** 10.1007/s00484-016-1254-4

**Published:** 2016-10-08

**Authors:** Danuta Stępalska, Dorota Myszkowska, Leśkiewicz Katarzyna, Piotrowicz Katarzyna, Borycka Katarzyna, Chłopek Kazimiera, Grewling Łukasz, Kasprzyk Idalia, Majkowska-Wojciechowska Barbara, Malkiewicz Małgorzata, Nowak Małgorzata, Piotrowska-Weryszko Krystyna, Puc Małgorzata, Weryszko-Chmielewska Elżbieta

**Affiliations:** 1 Institute of Botany, Jagiellonian University, Kraków, Poland; 2 Department of Clinical and Environmental Allergology, Jagiellonian University Medical College, 31-531 Kraków, Śniadeckich 10 Poland; 3 Institute of Information and Library Science of the Jagiellonian University, Kraków, Poland; 4 Institute of Geography and Spatial Management, Jagiellonian University, Kraków, Poland; 5 Department of Environmental Biology, University of Rzeszów, Rzeszów, Poland; 6 Faculty of Earth Sciences, University of Silesia, Sosnowiec, Poland; 7 Laboratory of Aeropalynology, Faculty of Biology, Adam Mickiewicz University, Poznań, Poland; 8 Department of Immunology, Rheumatology and Allergy, Medical University of Łódź, Łódź, Poland; 9 Laboratory of Paleobotany, Department of Stratigraphical Geology, University of Wroclaw, Wrocław, Poland; 10 Department of Dermatology, University of Medical Sciences, Poznań, Poland; 11 Department of General Ecology, University of Life Sciences in Lublin, Lublin, Poland; 12 Department of Botany and Nature Conservation, University of Szczecin, Szczecin, Poland; 13 Department of Botany, University of Life Sciences in Lublin, Lublin, Poland

**Keywords:** *Artemisia*, *Ambrosia*, Pollen season co-occurrence, Aerobiological monitoring, Poland

## Abstract

The Asteraceae family is one of the largest families, comprising 67 genera and 264 species in Poland. However, only a few genera, including *Artemisia* and *Ambrosia* are potential allergenic sources. The aim of the study was to estimate how often and to what degree *Artemisia* and *Ambrosia* pollen seasons co-occur intensifying human health risk, and how synoptic situations influence frequency of days with high pollen concentrations of both taxa. *Artemisia* and *Ambrosia* pollen data were collected, using the volumetric method, at 8 sites in Poland. Daily concentrations of *Artemisia* pollen equal to 30 grains or more and *Ambrosia* pollen equal to 10 grains or more were accepted as high values. Concentrations of more than 10 pollen grains were defined as high in the case of *Ambrosia* because its allergenicity is considered higher. High concentrations were confronted with synoptic situations. Analysis was performed on the basis of two calendars on circulation types of atmosphere in Poland (Niedźwiedź, 2006, 2015). Co-occurrence of *Artemisia* and *Ambrosia* pollen seasons is being found most often, when *Ambrosia* pollen season starts in the first half of August. If it happens in the last 10 days of August high pollen concentrations of *Artemisia* and *Ambrosia* do not occur at the same days. At three sites (Sosnowiec, Rzeszów, Lublin) high *Ambrosia* pollen concentrations during the *Artemisia* pollen season appear more often than in other sites under question. The high *Artemisia* pollen concentrations occur, when continental or polar maritime old air masses inflow into Poland. The impact of air masses on high *Ambrosia* pollen concentrations depends on site localizations. It is likely, that in the south-eastern part of Poland high *Ambrosia* pollen concentrations result from the pollen transport from east-south-south-westerly directions and the local sources. Co-occurrence of both taxa pollen seasons depends on the air masses inflow and appears more often in a south-eastern part of Poland.

## Introduction

The Asteraceae family is one of the largest families of plants, comprising approximately 1100 genera and 20,000 species including 67 genera and 264 species in Poland. However, only a few genera, including *Artemisia* (mugwort) and *Ambrosia* (ragweed) among others, are potential sources of allergenic pollenprovoking allergic rhinitis and conjunctivitis (D’Amato et al. 2007; Gadermaier et al. 2004; Peternel et al. 2008).

The genus of *Artemisia* comprises about 400 species and is widely distributed in temperate and humid zones of the northern hemisphere and along the Mediterranean basin. The most common *Artemisia* species in Europe are *A. vulgaris* L. (mugwort), *A. campestris* L. and *A. absinthium* L. (Tutin 1972). Other *Artemisia* species are also widespread in the central and eastern part of Europe including Poland, Hungary and Bulgaria, e.g.: *A. pontica* L., *A. annua* L., *A. maritima* L. and *A. scoparia* W. et K. (Soó 1970; Tutin 1972). *Artemisia* species colonizes disturbed soils in urban and rural habitats, roadside verges, agricultural fields and deserted places (Spieksma et al., 2003). *Artemisia vulgaris* occurs evenly throughout Poland. *Artemisia campestris* and *A. absinthium* have similar distribution to *Artemisia vulgaris* (Zając and Zając 2001).

The incidence of allergic diseases caused by *Artemisia* pollen in Europe is being estimated between 3 % and 15 % of pollinosis patients (D’Amato et al. 1998; Stach et al. 2007). It is known that significant cross-reactivity among ragweed species within the *Ambrosia* genus and also between the major allergens of *Ambrosia* and *Artemisia* appears (Dahl et al., 1999; Jäger, 2000). According to Spieksma (1986), 3–10 % of all all pollen sufferers are allergic to mugwort antigens and simultaneously allergic to ragweed and grass pollen allergens, and the allergens of apple and celery (Hirschwehr et al. 1998). Asero et al. (2006) studying *Artemisia* and *Ambrosia* hypersensitivity indicated that only 7 % of mugwort hypersensitive patients were not sensitized to ragweed, whereas 62 % of ragweed hypersensitive patients were not sensitized to mugwort. In Italy the threshold concentration of *Artemisia* pollen, which reveals clinical symptoms of pollinosis in allergic people, is reported by Voltolini et al. (2000) as 12 PG/m^3^ . In Poland the first symptoms of sensitization to *Artemisia* pollen emerge when the daily concentration amounts 30 PG/m^3^. The concentration of 70 PG/m^3^ provokes intensive symptoms (Rapiejko et al. 2007).

The genus of *Ambrosia* is composed of about 40 species of which only five have been recorded in Europe: *Ambrosia artemisiifolia* L. = *A. elatior* (short or common, annual ragweed), *Ambrosia trifida* L*.* (great or giant ragweed), *Ambrosia psilostachya* Torr. et Grey = *A. coronopifolia* (perennial ragweed)*, Ambrosia tenuifolia* Sprang. (silver ragweed) (Hansen 1976). However, short ragweed is the most widely spread of all (Járai-Komlódi and Juhász 1993). In Poland, the most frequently found species is *Ambrosia artemisiifolia* L. Currently this species occurs most commonly in the western and southwestern parts of Poland.

**Fig. 1:**
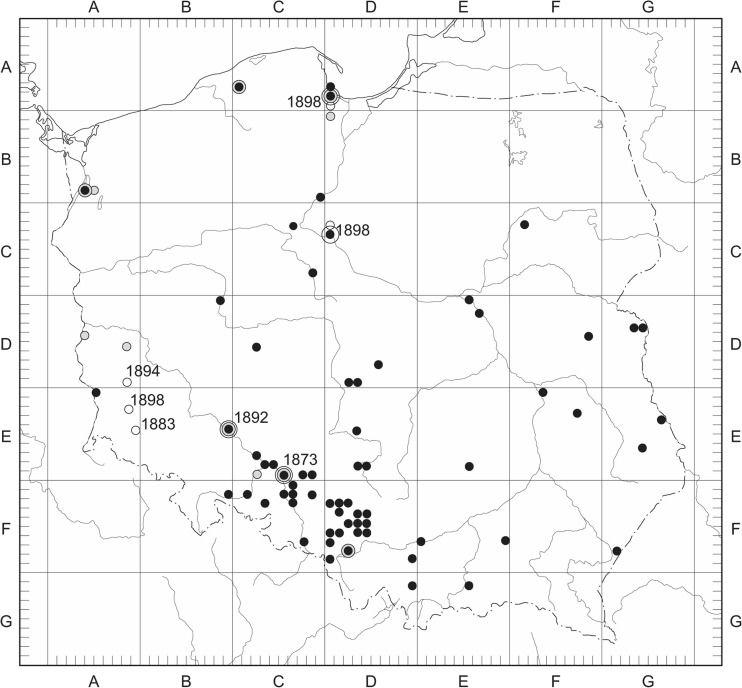
Recorded history of the spread of *Ambrosia artemisiifolia* L. in Poland drawn for the consecutive time periods.  between 1851 and 1900; ● between 1901 and 1950; ○ between 1951 and 2013. Updated map published by Tokarska-Guzik et al. (2011), given after Tokarska-Guzik approval

The historical spread of *Ambrosia artemisiifolia* in Poland is recorded in consecutive time periods (Fig. 1) (Tokarska-Guzik et al. 2011). *Ambrosia maritima* L. occurs in the Mediterranean region and is the only native species in Europe. The other four species are native to eastern and central North America from where they were imported as ballast weed to Europe. *Ambrosia* has evolved in response to a dry climate and open areas. In Europe *Ambrosia* has already become established mainly due to the large production of seeds that may remain dormant at least 39 years if conditions for germination are unsuitable and allow for its easy and rapid spread (Smith et al. 2013 and references therein). *Ambrosia* also produces allergenic pollen in enormous amounts: a single plant can produce millions of pollen grains that are small (18–22 μm) and can easily become airborne. They are considered to be one of the most potent allergens known (Comtois 1998; Weryszko – Chmielewska and Piotrowska 2008). The presence of ragweed in Europe was recorded for the first time at the beginning of the nineteenth century, although it became a real threat after the First World War (Juhász 1998). The places most contaminated with ragweed are Hungary, Croatia and parts of France, but it is also spreading in northern Italy, Switzerland, Austria, the Czech Republic, Slovakia and Bulgaria (Clot 2003; Smith et al. 2013 and references therein). The spread of ragweed seems to be limited by climate even if the human environment would allow its establishment (Comtois 1998; Saar et al. 2000). In areas with maritime climate, ragweed population does not appear to thrive and in Northern Europe the growing season is too short for seed maturation. Populations rely on the introduction of seeds from outside sources (Comtois 1998; Dahl et al. 1999). In Poland common ragweed colonizes cultivated fields and ruderal habitats, grows on disturbed soils, roadsides, near cereal elevators and harbours.

According to clinical experience, ragweed pollen appears to induce asthma approximately twice as often as it occurs in other pollen allergies (Gadermaier et al. 2004). In the area of Milan sensitization rates increased from 20 % to more than 60 % during a five year period (Asero 2002). Research in Austria stated that the higher *Ambrosia* pollen concentration, the higher the amount of ragweed pollen antibodies in the blood of patients suffering from inhalant allergy (Jäger and Litschauer 1998).

It should be stressed that different studies reported various threshold values of *Ambrosia* pollen concentrations provoking the first symptoms in sensitized patients. In France the threshold value is 5 pollen grains per m^3^ (PG/m^3^ ) (Dechamp et al. 1997) or 13 PG/m^3^ (Laaidi and Laaidi 1999), in Canada 5 PG/m^3^ (Comtois and Gagnon 1998), in Austria 20 PG/m^3^ (Jäger 1998), in Hungary 30 PG/m^3^ (Makra et al. 2005).

A knowledge that *Artemisia* and *Ambrosia* pollen seasons often overlap, provides information for estimating clinical and prophylactic aspects relating to cross-reactivity and co-sensitization. Studies performed hitherto discussed pollen seasons of these taxa separately, therefore the aim of our study was to estimate how often and to what degree *Artemisia* and *Ambrosia* pollen seasons co-occur and potentially intensify the risk to human health at eight selected sites in Poland, and to estimate the impact of specific synoptic situations on *Ambrosia* and *Artemisia* pollen concentrations.

## Materials and methods

### Study sites

**Fig. 2:**
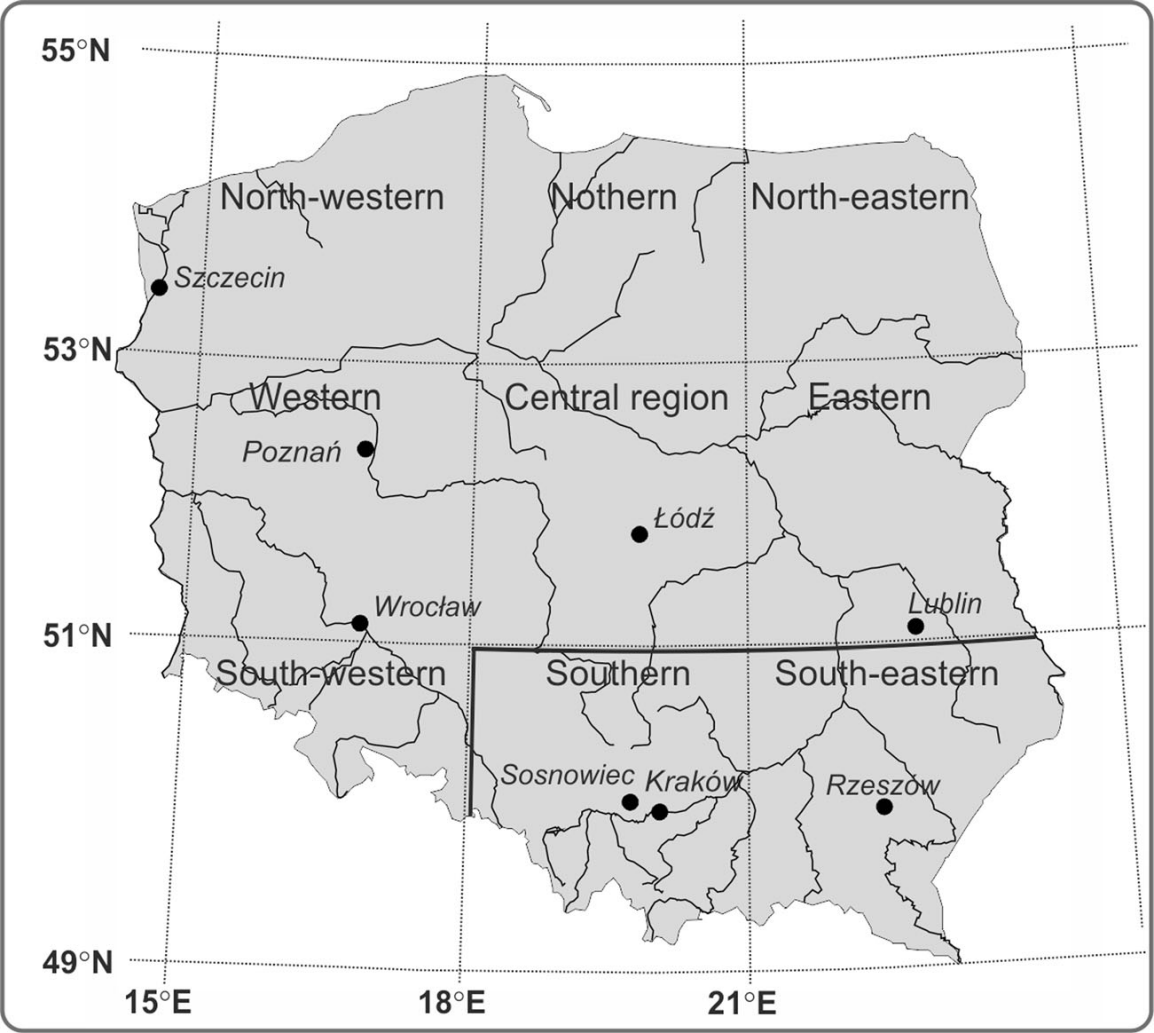
Map of Poland showing the monitoring sites according to regions classified by Niedźwiedź (2006). Two regions: southern and southeastern were marked with black, bold line as regions where air circulations, air masses and atmospheric fronts were classified (Niedźwiedź 2015)

*Ambrosia* and *Artemisia* pollen concentrations were analysed from eight selected sites in Poland (Szczecin, Poznań, Wrocław, Łódź, Sosnowiec, Kraków, Rzeszów, Lublin) (Fig. 2, Table 1). Poland is most frequently influenced by the polar-maritime (Pm) air masses originating from over the Northern Atlantic that bring thaw, an increase in cloudiness and snow in winter, and as well as chilling, an increase in cloudiness and rainfall in summer. The second most frequent air masses influencing Poland are polar-continental air masses (PPk), that bring warm, sunny and dry weather in summer and frosty weather in winter. The inflow of other air masses (arctic and tropical) is very seldom about 2–4 % of days during a year. The highest rainfall level is recorded in summer (June, July and August). In the annual cycle about 40 % of rain falls in these months (Dynowska 1991). According to Paszyński and Niedźwiedź (1991) the atmospheric circulation influences decisively climate in Poland. It causes the increase in continental features of climate in the eastern part of the country, and great variability of weather in short time periods. Therefore the climate in Poland is defined as transitional climate (Kożuchowski, 1999).

**Table 1:** Some information on study sites, including geographical location (coordinates, height above sea level and height above ground level) and the study period

Study site	Longitude (E)	Latitude (N)	Altitude a.s.l. (m)	Altitude a.g.l. (m)	Study period
Szczecin	14^o^ 33’	53^o^ 26’	52	21	2000–2012 (13 yrs)
Poznań	16^o^ 53’	52^o^ 24’	65–92	53	1996–2012 (17 yrs)
Wrocław	17^o^ 01’	51^o^ 06’	105–155	20	2002–2012 (11 yrs)
Łódź	19^o^ 28’	51^o^ 47’	150	15	2003–2012 (10 yrs)
Sosnowiec	19^o^ 08’	50^o^ 17’	263	20	1998–2012 (15 yrs)
Kraków	19^o^ 59’	50^o^ 04’	220	20	1992–2012 (21 yrs)
Rzeszów	22^o^ 02’	50^o^ 01’	200–215	12	1997–2012 (16 yrs)
Lublin	22^o^ 32’	51^o^ 14’	197	18	2001–2012 (12 yrs)

### Aerobiological data

The shortest aerobiological records used in our study were in Łódź (10 years) and the longest in Kraków (21 years). The aerobiological measurements were performed using volumetric spore traps of the Hirst design (Hirst 1952). All the traps were installed on roof tops at different heights above ground as seen in Table 1. The different sampling heights depended on the availability of buildings, where traps could be placed. Air was sucked into the trap at rate of 10 l/min through a 2 mm × 14 mm orifice flowing over a rotating drum that moved at 2 mm/h and which was coated with an adhesive, transparent tape. Pollen grains were sampled continuously. The tape used for catching pollen grains was replaced every week at the same day and cut into segments corresponding to 24 h periods. Segments were scanned and counted using a light microscope at 400 x magnification.

Daily average pollen concentrations are expressed as the number of pollen grains per cubic meter of air (PG/m^3^). To define the start, the end and the duration of the season the 90 % method was applied to eliminate long tails of low values at the start and the end of the seasons that could distort the limits of the seasons. The start of the season was defined as the date when 5 % of the seasonal cumulative spore count was trapped and the end of the season as the date when 95 % of the seasonal cumulative spore count was reached (Nilsson and Persson 1981). To compare the pollen concentrations over the studied years, a Seasonal Pollen Index (SPI) was calculated as the sum of the daily pollen counts in a given season. Because of the high allergenicity of *Ambrosia* pollen and as airborne pollen concentrations at some sampling sites sometimes marginally exceeded 10 grains per cubic meter of air, we accepted the value of 10 pollen grains m^−3^ as the threshold for ‘high’ concentration.

### Meteorological data

The influence of synoptic situations on *Artemisia* and *Ambrosia* pollen concentrations in the air was examined on the basis of two calendars on circulation types of atmosphere in Poland (Niedźwiedź 2006, 2015).

The type of the synoptic situation, air masses and atmospheric front in a given day was defined (http://klimat.wnoz.us.edu.pl). Classification by Niedźwiedź (2015) for the southern part of Poland could have been applied for the region within coordinates of 49-51^o^N and 18-25°E (Niedźwiedź 2015) (Fig. 2). For the remaining four sites, beyond these limits, other classification also by Niedźwiedź (2006) was used for 9 regions (Fig. 2). Unfortunately this classification was not available before 2001 and comprises only the type of air circulation without air masses and atmospheric fronts. According to this classification Łódź is situated in the central region, Wrocław and Poznań in the western region, and Szczecin in the north-western regions. The symbols of synoptic situation types are the same in both classifications (Table 2).

**Table 2:** Synoptic situations, air masses and atmospheric fronts (according to T. Niedźwiedź catalogue, 2014)

**Symbols**	**Circulation types**
E + SEa	Anticyclonic situation with an advection of air masses from East and South-East
Ca + Ka	Central anticyclonic situation, anticyclonic wedge
Cc + Bc	Central cyclonic situation, through of low pressure
S + SWc	Cyclonic situation with an advection of air masses from South and South-West
S + SWa	Anticyclonic situation with an advection of air masses from South and South-West
E + SEc	Cyclonic situation with an advection of air masses from East and South-East
W + NWc	Cyclonic situation with an advection of air masses from West and North-West
W + NWa	Anticyclonic situation with an advection of air masses from West and North-West
N + NEa	Anticyclonic situation with an advection of air masses from North and North-East
N + NEc	Cyclonic situation with an advection of air masses from North and North-East
x	Unclassified situation
	**Air masses**
PPk	Polar continental
PPms	Polar maritime old (transformed)
PPmc	Polar maritime warm
PPm	Polar maritime (fresh)
rmp	Various air masses in day
PZ	Tropical air masses
PA	Arctic air masses
	**Atmospheric fronts**
−	Day without front
z	Cold front
c	Warm front
st	Stationary front
r	Several various fronts in day
o	Occluded front (occlusion)

Detailed analysis on the influence of synoptic situations on *Ambrosia* pollen concentrations in the southern part of Poland was achieved by using regressive trees (C&RT). It was therefore possible to define circulation types, air masses and atmospheric fronts, which favour high concentrations. The occurrence of these synoptic situations in the last phase of the *Artemisia* pollen season favours overlapping of *Artemisia* and *Ambrosia* pollen seasons. This method allows the exploration of a great number of data, to define a set of synoptic parameters that determine the division of a dependent variable (daily concentration of *Ambrosia* pollen) into separable subsets of differentiation.

## Results

### Descriptive statistics of pollen seasons

The season start dates and the peak concentration exhibit low variability for both taxa at all the sites, but there are lower coefficients for *Artemisia* which means that the season start of *Ambrosia* is more variable from year to year. The highest coefficients of variability for *Artemisia* occur for days with PG/m^3^ ≥ 30 (20.2 % - 75.0 %) and for *Ambrosia* it occurs for days with PG/m^3^ ≥ 10 (39.0 % -92.0 %) (Table 3). The Kruskal-Wallis test revealed that start dates of the *Ambrosia* pollen season and their duration do not differ among sites (*p* > 0.05). In the case of *Artemisia*, there are statistically significant differences among sites (Table 3).

**Table 3:** *Artemisia* and *Ambrosia* pollen season characteristics in the selected cities in Poland. The descriptive statistics were calculated on the basis of data series presented in Table 1 in a given city. The coefficient of variability (*V%*) was calculated on the basis of not rounded values, the values of arithmetic mean ($\overline{x}$) and standard deviation (*s*) are expressed as integers. At the bottom of the table the results of Kruskal-Wallis test

Study site	Statistics/ season characteristics	*Artemisia*			*Ambrosia*		
		Season start^1^	Season duration^2^	Days with PG/m^3^ ≥ 30^2^	Season start^1^	Season duration^2^	Days with PG/m^3^ ≥ 10^2^
Szczecin	$\overline{x}$	18–07	40	9	22–08	29	2
	*s*	11	11	6	6	15	2
	*V*%	5.5	27.7	67.5	2.6	51.3	88.3
Poznań	$\overline{x}$	25–07	32	13	19–08	30	2
	*s*	4	12	7	8	12	2
	*V*%	2.1	37.2	52.0	3.5	41.9	159.5
Wrocław	$\overline{x}$	27–07	28	13	18–09	31	3
	*s*	3	8	5	13	16	2
	*V*%	1.5	28.7	36.2	5.6	52.8	78.8
Łódź	$\overline{x}$	20–07	39	9	14–08	35	3
	*s*	5	9	6	6	8	2
	*V*%	2.7	22.0	72.3	2.4	23.3	147.7
Sosnowiec	$\overline{x}$	25–07	38	26	17–08	37	7
	*s*	4	14	9	8	14	3
	*V*%	1.8	35.9	64.0	3.4	38.1	49.1
Kraków	$\overline{x}$	26–07	32	7	15–08	32	3
	*s*	3	13	5	12	15	3
	*V*%	1.7	42.4	75.0	5.2	46.8	95.9
Rzeszów	$\overline{x}$	29–07	34	7	17–08	27	7
	*s*	3	14	5	5	13	3
	*V*%	1.2	40.5	67.5	2.1	46.2	41.1
Lublin	$\overline{x}$	23–07	41	20	17–08	38	6
	*s*	4	13	4	6	14	2
	*V*%	1.8	31.5	20.2	2.8	35.5	42.0
Kruskal-Wallis test		*p* < 0.01	p < 0.01	p < 0.01	*p* = 0.18	*p* = 0.38	p < 0.01

Season characteristics were calculated using the 90 % method; $\overline{x}$ - arithmetic mean; *s* - standard deviation; *V*% - coefficient of variation

^1^date; ^2^number of days

### Overlapping of pollen seasons of both taxa

**Fig. 3:**
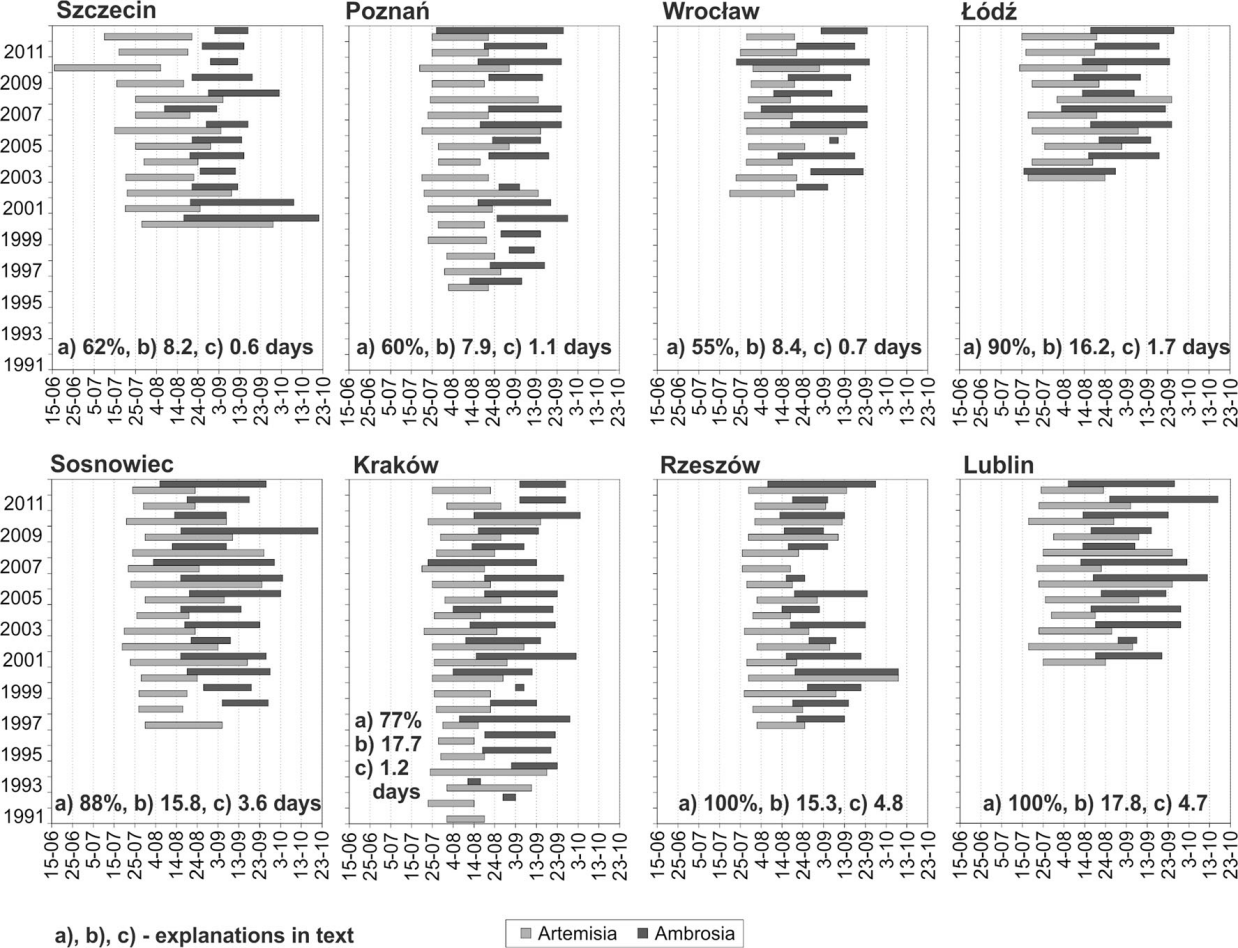
Start, end and duration of *Artemisia* and *Ambrosia* pollen seasons in given monitoring sites. a) percentage of days in the *Ambrosia* pollen seasons overlapping the *Artemisia* pollen seasons, b) number of overlapping days, c) average number of overlapping days with high pollen concentrations of both taxa

The *Artemisia* pollen seasons most often begin, in the second half of July at all the sites and start dates fluctuate in relatively small ranges from year to year. The most stable start dates of the *Artemisia* pollen season were recorded in Rzeszów (± 7 days) and the most variable in Łódź (± 18 days) (Fig. 3). The beginning of *Ambrosia* pollen seasons is much more variable from year to year. It most often falls in the second decade of August although the earliest start could have been in the middle of July (Łódź), and the latest in first days of September (Poznań, Sosnowiec, Kraków). Start dates of the *Ambrosia* pollen season varied the least in Szczecin, Rzeszów and Lublin and varied the most in Kraków and Wrocław.

**Fig. 4:**
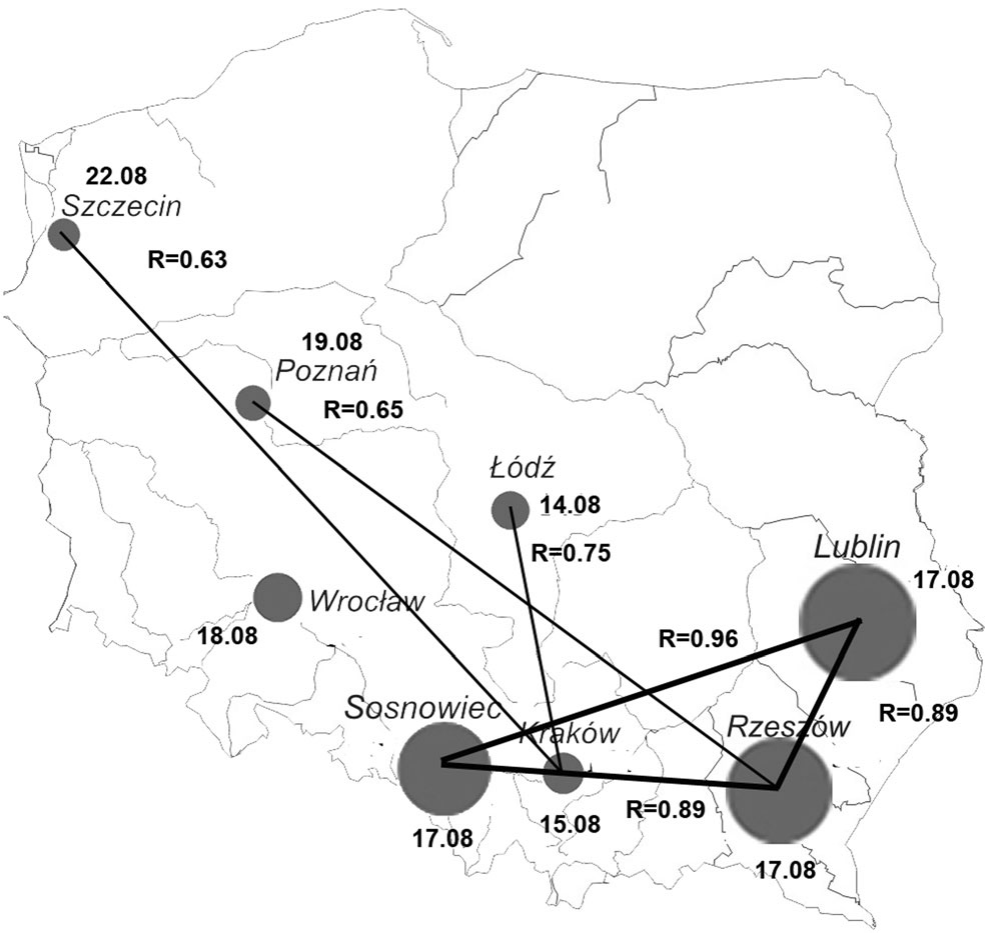
Selected characteristics of the *Ambrosia* pollen season: average date of the season start, average SPI (the bigger the circle the higher the SPI), correlation coefficient (R) between dates of season starts

The Spearman’s rank correlation test revealed that there were no statistically significant correlations between start dates of *Artemisia* and *Ambrosia* pollen seasons. It is likely, that the beginning of the *Ambrosia* pollen season is associated with inflow of air masses. This is confirmed by synchronization of pollen season start dates at three sites in southern Poland (Sosnowiec, Rzeszów, Lublin). The correlation coefficient between the start dates of *Ambrosia* pollen season at these sites is over 0.88 (Fig. 4).

The end of *Artemisia* and *Ambrosia* pollen seasons varied greatly In case of *Artemisia* the difference between the earliest and the latest end dates at all the sites was from 27 days in Łódź to 54 days in Szczecin and for *Ambrosia* it was from 20 days in Wrocław to 49 days in Szczecin (Fig. 3). Analysis revealed that the *Artemisia* pollen season usually started between 19 and 25 days before the beginning of the *Ambrosia* pollen season at the majority of sites. Figure 3 demonstrates that pollen seasons of both taxa could overlap at all sites although not every year. The values present in Fig. 3: (a) percentage of days in the *Ambrosia* pollen season overlapping the *Artemisia* pollen season, (b) number of overlapping days, (c) average number of overlapping days with high concentrations of both taxa.

**Fig. 5:**
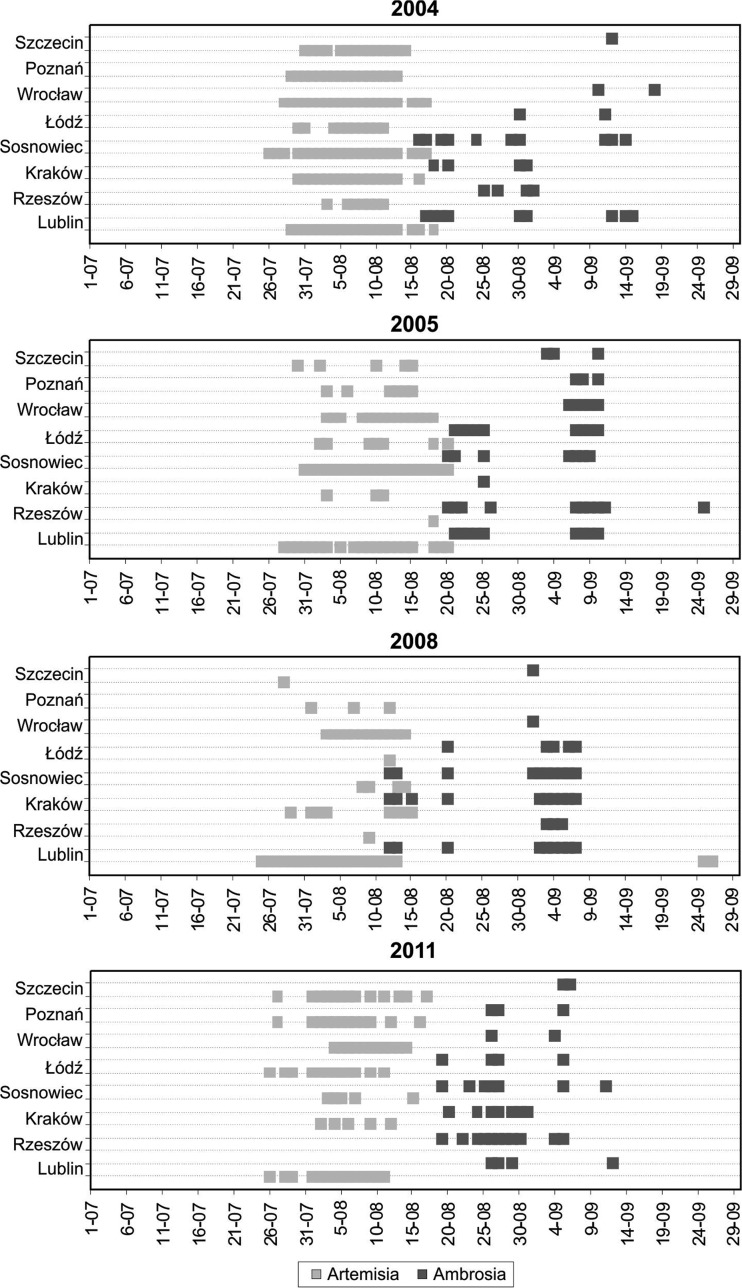
Time series of high *Artemisia* pollen concentrations (≥ 30 PG/m^3^) and *Ambrosia* pollen concentrations (≥ 10 PG/m^3^) in studied sites in selected years

Time series of high *Artemisia* pollen concentrations (≥30 grains) and high *Ambrosia* pollen concentrations (≥10 grains) presented in Fig. 5 show that the co-occurrence of high daily pollen concentrations of these taxa (single days or series of 2–4 days) most often occur in Sosnowiec and Lublin, and sporadically in Wrocław and Kraków. High daily *Artemisia* and *Ambrosia* pollen concentrations on these days could be a threat to allergic population*.* Such co-occurrence usually happens in August when the *Ambrosia* pollen season starts in the first half of August (2008). If the *Ambrosia* pollen season begins a little bit later, in the third decade of August, high pollen concentrations of *Artemisia* and *Ambrosia* do not occur on the same days (2011).

### Days with different ambrosia t pollen concentrations during the Artemisia pollen seasons

**Fig. 6:**
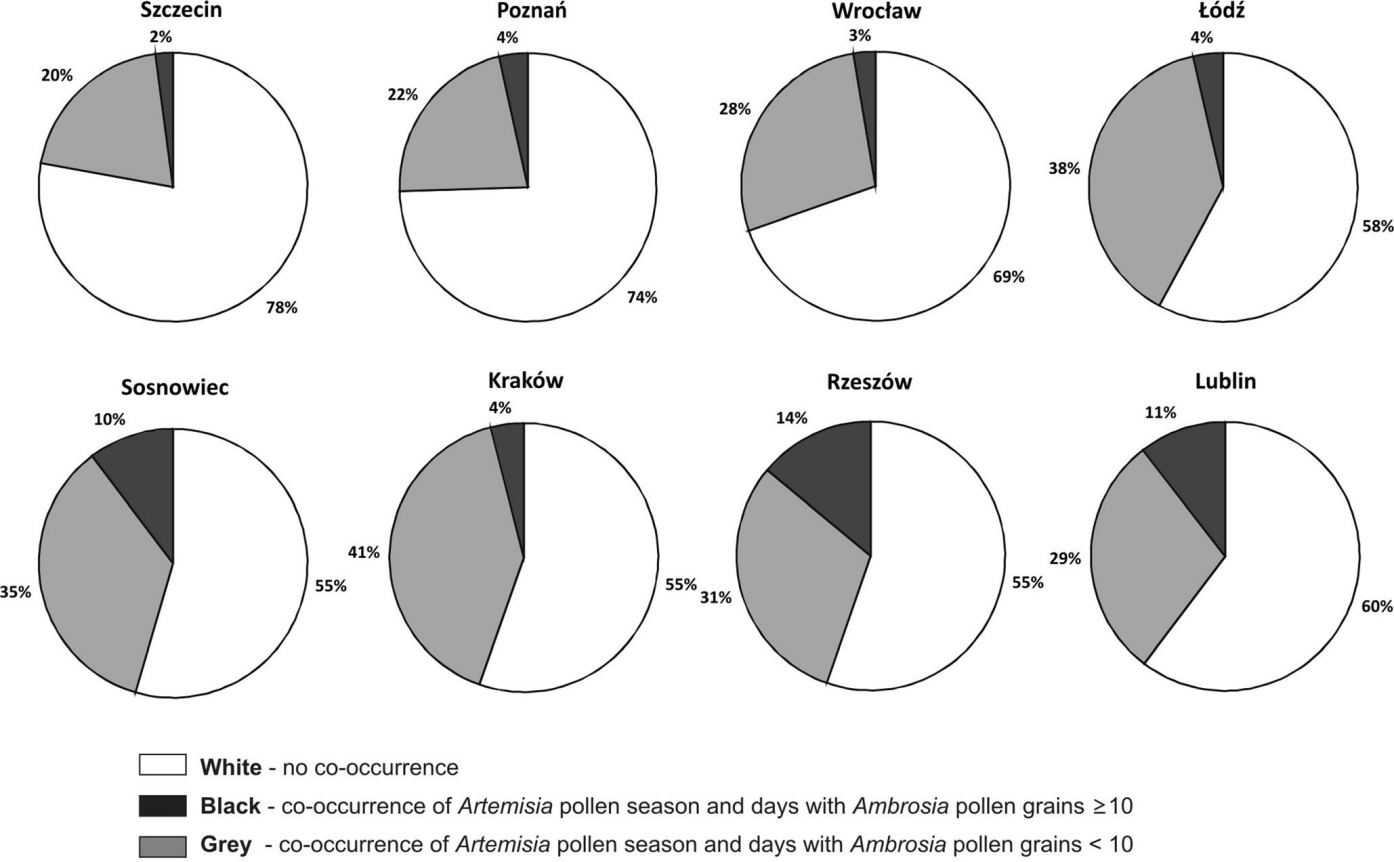
Pie chart of *Ambrosia* pollen occurrence during the *Artemisia* pollen seasons, where the whole circle (100 %) is the *Artemisia* pollen season

There are situations during *Artemisia* and *Ambrosia* pollen seasons when seasons of both taxa do not overlap at all. There are also situations when days with *Ambrosia* pollen grains equal to 10 PG/m^3^ and over 10 PG/m^3^ co-occur with the *Artemisia* pollen season. Days with *Ambrosia* pollen grains below 10 PG/m^3^ also show up, overlapping the *Artemisia* pollen season (Fig. 6). Looking at all sites, and assuming that the whole circle is the *Artemisia* pollen season (100 %), two groups of sites could be distinguished: sites, where the threat for sensitive people is low, below 5 % (Szczecin, Poznań, Wrocław, Łódź, Kraków) and the second group, where the threat is higher, over 5 % (Sosnowiec, Rzeszów, Lublin) (Figs 3 and 6).

### Days with high pollen concentration of both taxa in given synoptic situations

**Fig. 7:**
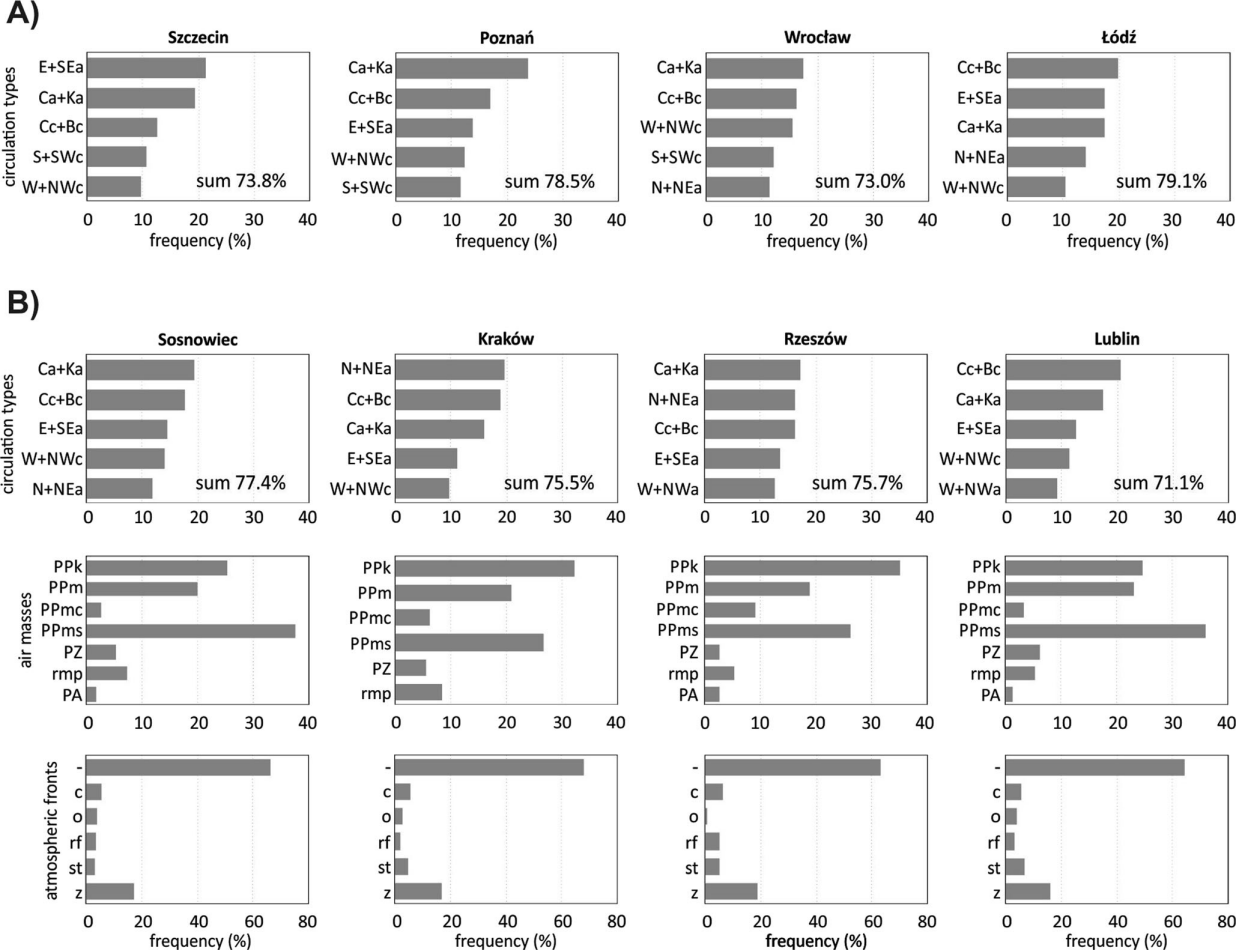
Frequency of days with high *Artemisia* pollen concentrations (≥ 30 PG/m^3^) in studied sites in given synoptic situations: A – in air circulation types; B – in air circulation types, air masses, atmospheric fronts (according to Niedźwiedź, T., catalogue, 2015)

High *Artemisia* pollen concentrations were examined in relation to synoptic situations: with air circulation types in all the sites and also with air masses and atmospheric fronts in case of sites in the southern part of Poland (Sosnowiec, Kraków, Rzeszów, Lublin) . Analysis of the frequency of days with high *Artemisia* pollen concentrations in different air circulation types indicates no clear relationship. High pollen concentrations (≥ 30 PG/m^3^ ) appeared with similar frequency (10–20 %) in no advection situation (central anticyclone situation - Ca, anticyclonic wedge – Ka, central cyclone situation – Cc, through of low pressure – Bc) and in other situations (Fig. 7). The relationship between high pollen concentration and air masses was more distinct. High pollen concentrations occurred when polar continental (PPk) or polar maritime transformed (PPms) air masses were over the studied sites. These air masses bring warm, even hot and dry weather with no precipitation and no atmospheric fronts. For Szczecin, Poznań, Wrocław and Łódź it is difficult to point out a type of situation, that could be decisive for high *Artemisia* pollen concentration. In these sites pollen concentration in a given day seems to be dependent on the air masses rather than on the air circulation type. The increase in *Artemisia* pollen takes place when the weather is warm, dry, no precipitation. Such situation is provoked by polar continental or polar maritime transformed air masses.

**Fig. 8:**
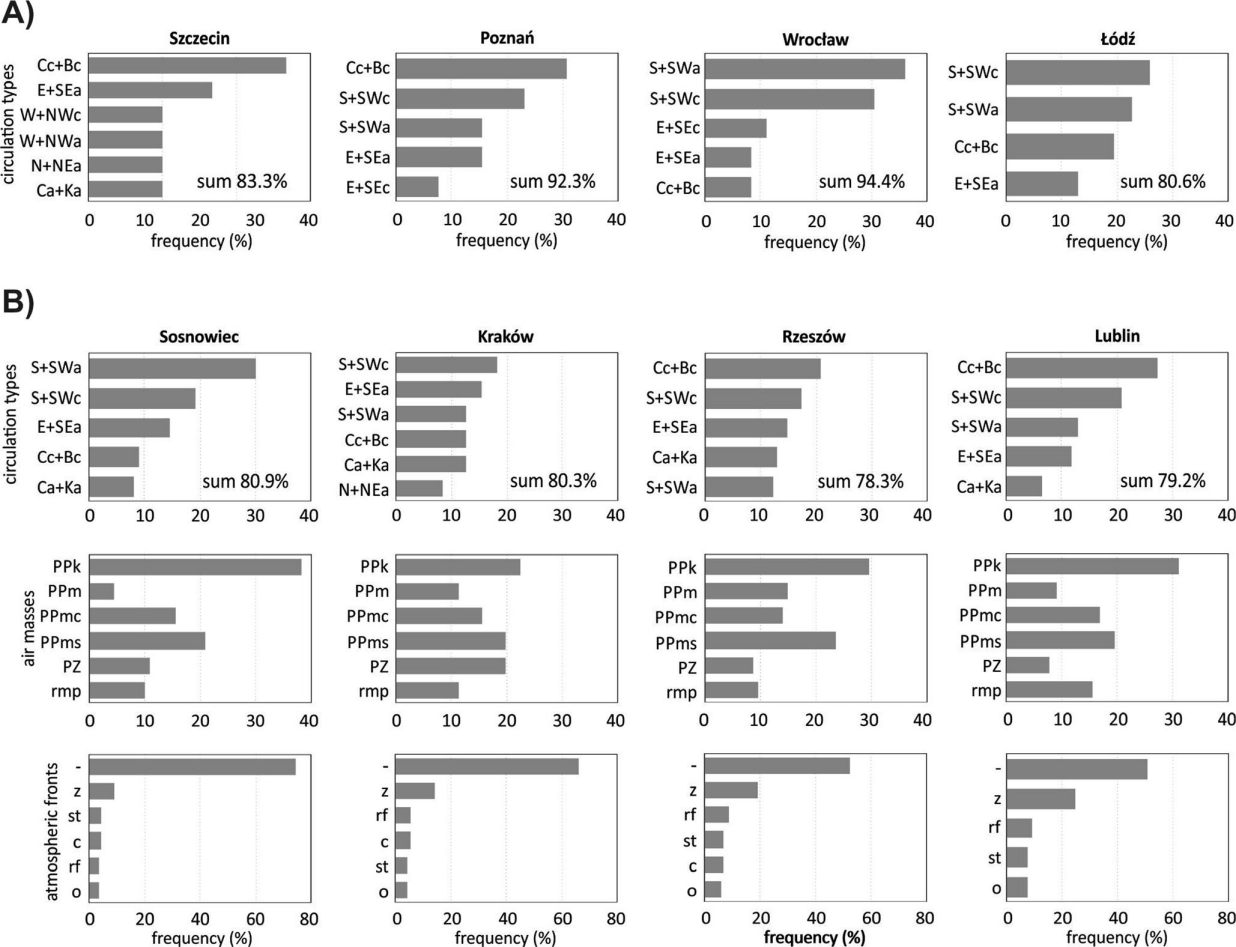
Frequency of days with high *Ambrosia* pollen concentrations (≥ 10 PG/m^3^) in studied sites in given synoptic situations: A – in air circulation types; B – in air circulation types, air masses, atmospheric fronts (according to Niedźwiedź, T., catalogue, 2015)

The regressive trees (C&RT) and analysis of frequency of days with high *Ambrosia* pollen concentrations (≥10 PG/m^3^ grains) showed results different than in the case of *Artemisia*. It was stated that in Rzeszów and Lublin days with high pollen concentrations occurred most often when southern Poland was influenced by low pressure, especially by central cyclonic (Cc) or through of low pressure (Bc) and cyclonic advection from south or south-west directions (S + SWc) (Fig. 8). In Sosnowiec and Kraków high pollen concentrations were associated mainly with air advection from south and south-west directions (S + SW) or east and south-east directions (E + SE) no matter what situation, cyclonic or anticyclone was. Generally high *Ambrosia* pollen concentrations accompanied polar continental (PPk) and polar maritime transformed (PPms) air masses, and in Kraków also tropical air masses advection (PZ). Days with high pollen concentrations occurred most often when no atmospheric fronts were present. In Wrocław and Łódź, similarly as in Sosnowiec and Kraków high *Ambrosia* pollen concentrations were associated with air advection from south and south-west directions (S + SWa or S + SWc) no matter what situation, cyclonic or anticyclonic was. In Szczecin and Poznań the frequency of days with high pollen concentrations was similar to that in Rzeszów and Lublin, when low pressure dominated (Fig. 8).

## Discussion

Analysis of *Artemisia* and *Ambrosia* pollen season characteristics revealed only two characteristics, a season start date and a day of maximum concentration with the low coefficient of variability for both taxa in all sites. Similar result, on the low coefficient of variability for the *Ambrosia* pollen season start, was given by Kasprzyk (2008). It could result from time during the year (July, August) when both taxa start their pollen seasons. Generally, the value of the coefficient of variability depends on thermal conditions that are more stable in later months of the year (Myszkowska et al. 2011). The pollen season start of early spring taxa e.g. *Alnus* and *Corylus* demonstrate the highest seasonal variability which depends on changeable thermal conditions at the beginning of the year (Kasprzyk et al. 2004; Rodriguez-Rajo et al. 2004).

The pollen seasons of *Artemisia* begin, most often, in the second half of July at all the studied sites. Similar results were reported by Grewling et al. (2012) in their work on the variation of *Artemisia* pollen seasons in Central and Eastern Europe. They indicated that *Artemisia* pollen seasons generally occurred between the middle of July and September. These findings confirmed the earlier work by Spieksma et al. (1989), who stated that start dates did not vary very much at certain sites in Europe. *Artemisia* flowers in Central Europe at the end of July while in Mediterranean areas pollination occurs mainly in September. This delay results from releasing *Artemisia* pollen after the peak of summer temperature in the Mediterranean region or generally from the effect of rainfall, as even single day’s rain, sharply stops the pollination within the main pollen season, or higher nutrient availability (Spieksma et al. 1989; D’Amato and Spieksma 1990). Puc (2006) noted the start of mugwort in the third decade of July, which agrees with our study. As regards the *Ambrosia* pollen season start, it is much more variable and dependent on site. On sites where the pollen season starts were least differentiated it could have resulted from local sources.

In contrast to the low variability found for the season start day and the day of maximum pollen concentration, the values of *Ambrosia* and *Artemisia* seasonal pollen index are the most variable season characteristics. Over consecutively studied years the values of *Ambrosia* SPI in Szczecin, Poznań, Wrocław, Łódź and Kraków were relatively similar and lower in comparison with Sosnowiec, Rzeszów and Lublin where higher pollen concentrations could result from a long distance transport. These findings coincide with reports by Chłopek and Tokarska-Guzik (2006), Malkiewicz and Wąsowicz (2003), Piotrowska and Weryszko-Chmielewska (2006), Puc (2004) and Stępalska et al. (2002). Ragweed pollen is one of the most abundant pollen type in the Pannonian Plain such as Croatia, where yearly total sums exceed 24,000 grains (Ivanić Grad) (Peternel et al. 2005), which is many times higher than in Polish sites.

Considering the spatial distribution of sites in Poland, statistically significant differences only occurred in the case of *Artemisia* pollen season start dates and duration, while *Ambrosia* pollen season start dates and their durations only differed significantly at three sites in southern Poland. Differences in airborne pollen concentrations are due to variable wind directions and depend on the distance between the site of measurement and the source of emission (Emberlin and Norris-Hill 1991). Furthermore, the large-scale dispersion of atmospheric constituents is controlled by synoptic-, continental-, or hemispheric- scale meteorological phenomena (Sofiev et al. 2013). Pollen transport is affected by a combination of wind direction, wind speed and wind duration, assuming no major rainfall. Damialis et al. (2005) reported an important effect of the wind direction on airborne pollen concentrations, particularly in the case of numerous pollen sources lying in one direction. A similar dependence between pollen concentrations and the presence of pollinating taxa in the vicinity of the measurement site reported Arobba et al. (2000) in Genoa. In Szczecin high atmospheric pollen concentrations of ragweed and mugwort were recorded at the site in the vicinity of which there were numerous plants of these taxa (Puc 2006). Analyses of *Artemisia* pollen seasons in eight sites in Poland in 2001–2005 revealed the clear differences in annual pollen totals among sites and years (Weryszko-Chmielewska et al. 2006).

Co-occurrence of pollen seasons of different taxa was reported earlier by only few papers. The phenomenon of co-occurrence is often considered as a result from extension of pollen seasons caused by gradual global warming and then there is possibility of overlapping. Pollen seasons of species flowering in summer have become longer. The end of Poaceae, *Artemisia, Urtica* and *Ambrosia* pollen seasons has tended to appear later (Wan et al. 2002). The comparison of *Artemisia* and *Ambrosia* pollen seasonal dynamics in Cracow, Poland shows, that there is possibility of the high pollen concentration occurrence of both taxa simultaneously, in the second part of August (late summer). This could be the reason for the increase in allergy symptoms resulting from a cross-reaction between allergens of both taxa (Myszkowska et al. 2012). Moreover, in the case of the late summer pollinating plants (*Artemisia* and *Ambrosia*), the negative correlation between pollen season start and end was found, the later pollen season starts, the earlier pollen season ends (Myszkowska et al. 2011).

In aerobiological literature it is stressed that increased temperature during summer and early autumn could cause the increasing growth of *Ambrosia* plants, and increasing pollen production; and this phenomenon is associated often with the higher concentration of CO_2_ in the air (Smith et al. 2013 and references therein). Barnes et al. (2001) stated that temperature and relative humidity slightly influence daily *Ambrosia* pollen concentrations but passing cold fronts have the greatest effect on airborne *Ambrosia* pollen concentrations. According to studies on phenological phases it is stated that the length of the pollen season becomes extended in plants flowering in summer (Huynen et al. 2003) that could result in co-occurrence of different species.

According to our present results, cnalysis of *Artemisia* pollen concentrations against a background of synoptic situation revealed that there is no clear relationship between frequency of days with high pollen concentrations in different air circulation types. However, the relationship between high pollen concentration and air masses was sharply outlined. High pollen concentrations occurred when polar continental (PPk) from the east or polar maritime transformed (PPms) air masses from the west were over the studied sites which suggests the case of the regional transport and sporadically long-distance transport. Long distance transport (LDT) episodes of airborne pollen are usually irregular (Smith et al. 2008) and can modify the characteristics of the pollen season (Sofiev et al. 2006). LDT episodes have been earlier identified for *Ambrosia* (Sikoparija et al. 2009; Stach et al. 2007), *Artemisia* (Izquierdo et al. 2011) and *Betula* (Skjøth et al. 2007).

In Budapest, Hungary, significant differences might be found in *Ambrosia* pollen concentrations on days with different weather types. Most of the anticyclonic situations are favourable for pollination, and all cyclonic situations are unfavourable. However, a part of the anticyclonic types were also unfavourable (Fehér and Járai-Komlódi 1996). Analysis of wind directions occurring the most often in Cracow showed *Ambrosia* pollen concentrations associated to wind blowing from easterly (E) and east-southeasterly (ESE) directions which indicated the long distance transport from the Czech Republic, Slovakia, Hungary and Ukraine. Wind from westerly (W) and west southwesterly (WSW) directions could have brought pollen from local sources in the western part of Poland (Stępalska et al. 2008). Kasprzyk (2008) noted high airborne *Ambrosia* pollen concentrations recorded on days when air mass advection came from east and south east, and from south and south-west. On days with polar maritime or arctic air masses, pollen concentrations were statistically significantly lower than on days with polar continental, polar maritime warm and polar maritime transformed air masses. The study performed in Sosnowiec, Poland on the threat of *Ambrosia* pollen at a regional scale showed a high negative correlation between frequency of air masses (polar maritime) from the west and the annual sum of pollen grains and maximum daily concentration (Chłopek et al. 2011). Hot and dry weather on the Pannonian Plain (PP) favours the release *Ambrosia* pollen during the flowering season. Adequate synoptic situations are required for air masses bearing pollen to move northward causing LDT from the PP to Poland and further into Scandinavia (Šikoparija et al. 2013).

## Conclusions

1. The *Artemisia* pollen seasons most often start in the second half of July at all the sites, while *Ambrosia* pollen seasons starts are much more variable.
2. Season start dates and dates of maximum concentration for *Artemisia* and *Ambrosia* show the lowestcoefficient of variability.
3. Pollen seasons of both taxa could overlap at all the sites although not every year. In Lublin and Rzeszów (100 % each site), and in Łódź (90 %) the co-occurrence of both taxa pollen seasons is the highest, while in Szczecin (62 %), Poznań (60 %), and Wroclaw (55 %) is the lowest.
4. In south-eastern Poland (Sosnowiec, Rzeszów, Lublin) high *Ambrosia* pollen concentrations during the *Artemisia* pollen season appear more often than in other study sites. It is, on the average, 3–5 days in a year.
5. High *Artemisia* pollen concentrations occur when polar continental (PPk) air masses inflow into Poland from the east or when polar maritime transformed (PPms) air masses come from the west.
6. In case of *Ambrosia*, days with high pollen concentrations occurred most often when sites under question were affected by low pressure and cyclonic advection from south or south-west directions (Szczecin, Poznań, Rzeszów, Lublin) or high pollen concentrations were associated mainly with air advection from south and south-west directions or east and south-east directions irrespective of cyclonic or anticyclonic situation (Wrocław, Łódź, Sosnowiec, Kraków).
7. Co-occurrence of pollen seasons of both taxa depends on the type of atmospheric circulation.
